# Catenane Structures of Homoleptic Thioglycolic Acid-Protected Gold Nanoclusters Evidenced by Ion Mobility-Mass Spectrometry and DFT Calculations

**DOI:** 10.3390/nano9030457

**Published:** 2019-03-19

**Authors:** Clothilde Comby-Zerbino, Martina Perić, Franck Bertorelle, Fabien Chirot, Philippe Dugourd, Vlasta Bonačić-Koutecký, Rodolphe Antoine

**Affiliations:** 1 Institut Lumière Matière UMR 5306, Université Claude Bernard Lyon 1, CNRS, Univ Lyon, F-69100 Villeurbanne, France; clothilde.zerbino@univ-lyon1.fr (C.C.-Z.); franck.bertorelle@univ-lyon1.fr (F.B.); philippe.dugourd@univ-lyon1.fr (P.D.); 2 Center of Excellence for Science and Technology-Integration of Mediterranean region (STIM) at Interdisciplinary Center for Advanced Sciences and Technology (ICAST), University of Split, Poljička cesta 35, HR-21000 Split, Croatia; martina@stim.unist.hr (M.P.); vbk@stim.unist.hr (V.B.-K.); 3 Institut des Sciences Analytiques UMR 5280, Université Claude Bernard Lyon 1, ENS de Lyon, CNRS, Univ Lyon, 5 rue de la Doua, F-69100 Villeurbanne, France; fabien.chirot@univ-lyon1.fr; 4 Department of Chemistry, Humboldt Universitat zu Berlin, Brook-Taylor-Strasse 2, 12489 Berlin, Germany

**Keywords:** gold nanoclusters, thiolate, catenane, ion mobility, DFT calculations

## Abstract

Thiolate-protected metal nanoclusters have highly size- and structure-dependent physicochemical properties and are a promising class of nanomaterials. As a consequence, for the rationalization of their synthesis and for the design of new clusters with tailored properties, a precise characterization of their composition and structure at the atomic level is required. We report a combined ion mobility-mass spectrometry approach with density functional theory (DFT) calculations for determination of the structural and optical properties of ultra-small gold nanoclusters protected by thioglycolic acid (TGA) as ligand molecules, Au_10_(TGA)_10_. Collision cross-section (CCS) measurements are reported for two charge states. DFT optimized geometrical structures are used to compute CCSs. The comparison of the experimentally- and theoretically-determined CCSs allows concluding that such nanoclusters have catenane structures.

## 1. Introduction

Thiolate-protected metal nanoclusters (NCs) are a promising class of nanomaterials due to fascinating molecular-like properties along with well-defined molecular structures [1,2,3]. However, their physicochemical properties are highly size- and structure-dependent. As a consequence, for the rationalization of their synthesis and for the design of new clusters with tailored properties, a precise characterization of their composition and structure at the atomic level is required.

The structural features of stoichiometric Au_n_(SR)_n_ gold nanoclusters (SR:thiolate ligand) was predicted to change from single rings to interlocked ring motifs (i.e., catenane structures) when n ≥ 10 [4]. The interlocked ring motif is a unique feature of homoleptic [Au(I)-SR]_x_ complexes found in Au_10_(SR)_10_, Au_11_(SR)_11_, and Au_12_(SR)_12_ [5,6,7]. More importantly, the catenane-like staple motifs predicted for Au_15_(SR)_13_ and Au_24_(SR)_20_ suggest that, at a Au/SR ratio approaching 1/1, the interlocked staple motifs may become a widespread conformation in thiolate-protected metal nanoclusters [8,9,10]. Moreover, the Au_10_(SR)_10_ catenane structure was recently identified as the best structural candidate for the Au local structure in bovine serum albumin protein-stabilized gold nanoclusters [11]. We reported in a recent work, a “one-pot–one-size” synthesis of Au_10_(SG)_10_ NCs (SG:glutathione:γ-L-glutamyl-L-cysteinylglycine) characterized by electrospray MS. The X-ray diffraction pattern of Au_10_(SG)_10_ was utilized as fingerprints for homoleptic gold–glutathione catenanes [7]. Regarding optical properties, enhanced second harmonic response and circular dichroism signals in the spectral region of 250–400 nm were observed due to this catenane structure exhibiting a centrosymmetry-broken structure [7]. Recently, Chevrier et al. confirmed the catenane structure by using synchrotron-based X-ray absorption fine structure (XAFS) spectroscopy [11]. As a complement to these powder-based structural characterization techniques requiring X-ray beams or synchrotron facilities, mass spectrometry-based techniques performed on gas phase nanoclusters ions may provide information on 3D molecular structures. In particular, ion mobility spectrometry (IMS) has been used for the characterization of gas-phase ligand-protected metal nanoclusters [12,13,14,15,16,17,18,19]. IMS separation is based on the different velocities adopted by ions travelling in an inert gas under a low electric field. The drift time of the ions through the IMS tube depends on the ratio between their collision cross-section (CCS) with the gas and their charge, thus allowing isomer discrimination. Our groups showed how IMS studies can provide insight into the size of glutathione-protected gold nanoclusters, as well as in the structural determination of inorganic nanoclusters [16,18,19].

In a previous recent work, we reported an ion mobility-mass spectrometry (IM-MS) approach for the analysis of homoleptic Au_10-12_(SG)_10-12_ nanoclusters. CCS measurements were reported for different charge states for Au_10_(SG)_10_, Au_11_(SG)_11_, and Au_12_(SG)_12_ nanoclusters [18]. Strong charge-state effects on experimental CCS values were observed and attributed to charge-induced glutathione unfolding. However, the importance of core structure and the ligand conformations on the total CCS was difficult to disentangle due to conformational effects of such a flexible peptide ligand. The IMS technique was not sufficient to discriminate between different possible structures (in particular catenane structures) for the core.

This discrimination could be easier if smaller and more rigid ligands are used for protection, where charge-induced ligand unfolding effects will be minimized. In this case, the structural characterization of clusters may thus be possible by comparing the arrival time distribution profiles recorded by ion mobility mass spectrometry with theoretical calculations using molecular modelling (density functional theory, DFT) and subsequent collision cross-section calculations using projection approximation. Here, we report a combined ion mobility and spectrometry approach with DFT calculations for the analysis of a stoichiometric gold nanocluster ligated by thioglycolic acid Au_10_(TGA)_10_ (TGA; see Figure S1 in the Supplementary Materials) as ligand molecules. Collision cross-section (CCS) measurements are reported for two charge states. DFT calculations have been performed to optimize different candidate structures for which CCSs were computed. The comparison of the experimentally- and theoretically-determined CCSs allows concluding about the catenane structures of such nanoclusters.

## 2. Materials and Methods

***Materials and synthesis protocol:*** All the chemicals were commercially available and were used without purification. HAuCl_4_·3H_2_O, trifluoroacetic acid (TFA), and methanol (HPLC grade) were purchased from Carl Roth (Lauterbourg, France). Thioglycolic acid (TGA), NaOH, and NH_4_OH were purchased from Sigma-Aldrich (Saint-Quentin Fallavier, France). Milli-Q water with a resistivity of 18.2 MΩ cm^−1^ was used for all experiments. Au_10_(TGA)_10_ NC was prepared as described in [7] with TGA as the ligand instead of glutathione. Briefly, 70 mg of TGA (≈53 µL) were diluted in 35 mL of methanol and 2 mL of triethylamine. Then, 100 mg of HAuCl_4_·3H_2_O in 15 mL of water were added, and the solution was stirred overnight at ambient temperature. To induce precipitation, 2 mL of 1 M NaOH solution were added, and the solution was centrifuged (10 min at 11,000 rpm).

***Ion mobility-mass spectrometry:*** Ion mobility measurements were performed using an ion mobility spectrometer as described in [20]. Measurements were done using a fresh mixture of Au_10_(TGA)_10_, prepared in an aqueous solution to a concentration of about 50 µM and directly electrosprayed using a syringe pump. Mobility measurements were done by injecting ion bunches in the drift tube filled with 4.0 Torr helium, in which a constant drift field was maintained through the controlled voltage drop across the tube. The temperature of the whole instrument was kept at 296 K. After their drift, ions were transferred to a reflector time-of-flight mass spectrometer. Mass spectra were finally recorded as a function of the IMS drift time, allowing for extraction of arrival time distributions (ATDs) for ions with any desired mass-to-charge ratio. Collision cross-sections (CCS) were extracted from ATDs as described in [21]. Using this method, the error of the experimental CCS was estimated to be 2%.

***Computational:*** The structural and absorption properties of Au_10_(TGA)_10_ were determined using the DFT and its time-dependent version TD-DFT approach [22,23]. For gold atoms, a 19-electron relativistic effective core potential (19e-RECP) was employed [24]. The structural and spectroscopic properties of Au_10_(TGA)_10_ were obtained at the PBE0/Def2-SVP level of theory [25,26].

## 3. Results and Discussion

### 3.1. Characterization of Au_10_(TGA)_10_

**Figure 1:**
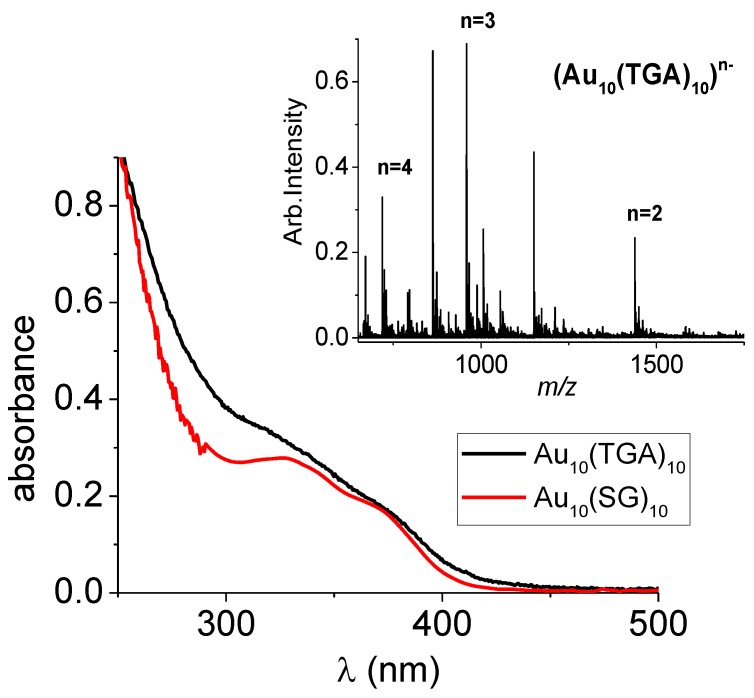
Experimental absorption spectra of Au_10_(SR)_10_ nanoclusters (NCs) (with SR = thioglycolic acid (TGA)and SG (see [7]). (Inset) Electrospray ionization ESI mass spectrum of the as-synthesized Au_10_(TGA)_10_ NCs.

The formation of Au_10_(TGA)_10_ NCs as the product was confirmed by electrospray ionization-mass spectrometry (ESI-MS) in negative mode (see the inset in Figure 1). A charge state distribution of the general formula [M−*n*H^+^]*^n^*^−^ (2 ≤ *n* ≤ 4) was observed for the Au_10_(TGA)_10_. The additional peaks observed in MS spectra were due to smaller stoichiometric (AuTGA)_n_ complexes (*n* ≤ 6) originating from the “in-source” fragmentation of the Au_10_(TGA)_10_ clusters (as evidenced by collision-induced dissociation experiments; see Figure S2 in the Supplementary Materials).

Concerning the optical properties, the one-photon absorption spectrum of the as-synthesized Au_10_(TGA)_10_ NCs showed a monotonic increase of intensity below 390 nm and a shoulder at ~310 nm. There was similarity with the absorption spectrum of the previously-reported Au_10_(SG)_10_ NCs (see Figure 1) [7].

### 3.2. Theoretical Investigation of the Structural and Optical Properties of Au_10_(TGA)_10_

**Figure 2:**
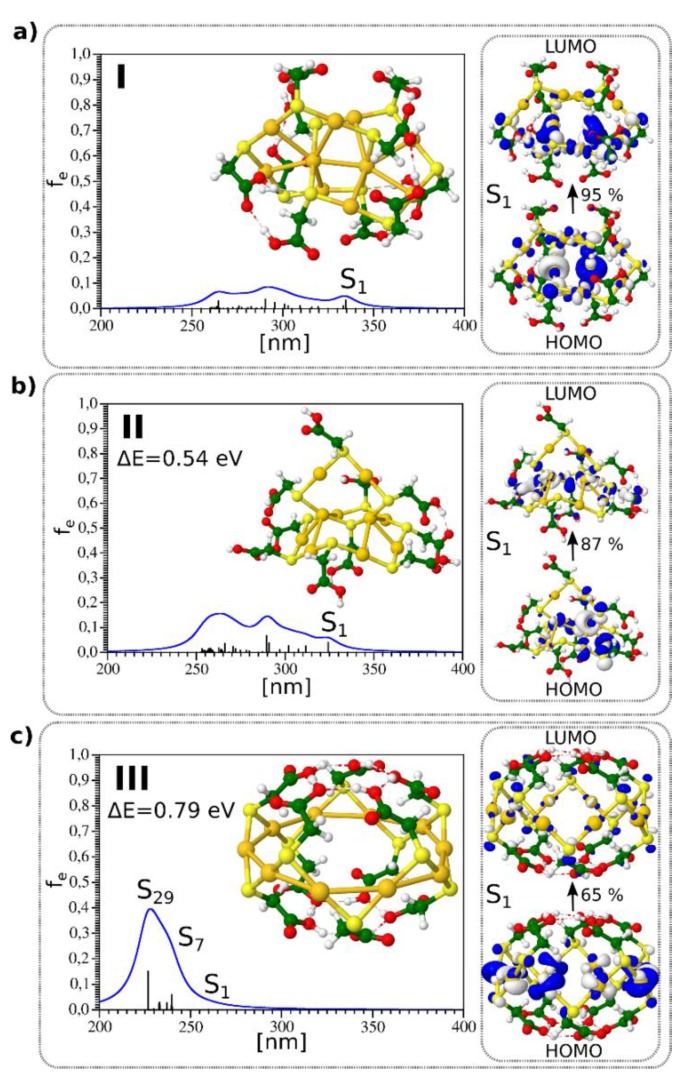
TD-DFT absorption spectrum and structure for three lowest energy isomers of Au_10_(TGA)_10_ shown in (**a**–**c**) respectively. Leading excitations responsible for the characteristic features of absorption are illustrated on the right side. HOMO-LUMO for isomers I, II, and III are 4.54, 4.62, and 5.55 eV, respectively.

The DFT method has been used to determine the structures of the Au_10_(SR)_10_ NCs based on the results obtained by a genetic algorithm search method [4]. The [5,5] catenane structure containing two interpenetrating −AuSR− pentagons was found to be the most stable structure (Figure 2a). The [6,4] structure containing four- and six-membered Au rings interpenetrating each other (Figure 2b) and the crown-like structure (Figure 2c) was higher in energy. The structure of these three isomers is shown in Figure 2. Interestingly, the size of TGA ligand along with the size of the crown and the Au-S bond length allowed for a rich hydrogen-bonding network within the TGA ligands, leading to a “ball-like” shape for the crown-like structure.

The absorption spectra calculated using a TD-DFT approach for the three isomers with catenane structures are also shown in Figure 2. For the [5,5] and [6,4] catenane structures, the first excited states were located between 320 and 350 nm. The leading excitations responsible for S_1_ and S_2_ excited states shown also in Figure 2 involved Au–Au aurophilic subunits bound to neighboring sulfur atoms and arose from the penetration of the two rings into each other. The absorption spectrum for the crown-like structure obtained from the TD-DFT approach differed considerably from those of other two isomers.

### 3.3. Catenane Structures of Homoleptic Au_10_(TGA)_10_ Evidenced by Ion Mobility-Mass Spectrometry and DFT Calculations

In order to characterize the structural properties of Au_10_(TGA)_10_ NCs, we conducted ion mobility-mass spectrometry (IM–MS) measurements. The extracted arrival time distributions (ATDs) were mainly monomodal for the two- and three-charge states of Au_10_(TGA)_10_, indicating that the corresponding clusters presented essentially a single structural type, and the width of the peaks was compatible with a single structural type being present (see Figures S3 and S4 in the Supplementary Materials). In addition, Figure S4 in the Supplementary Materials shows that the arrival time distributions (ATDs) for [Au_10_(TGA)_10_−2H]^2−^ and [Au_10_(TGA)_10_−3H]^3−^ were very close to the predicted ATDs by the Fick law. The observed ATDs peaks were thus limited by the experimental instrumental resolution. This means that the observed single peaks in ATDs corresponded to single structures, and other possible effects (conformational freedom and especially motion around the Au–S bond in the TGA ligand and possible interconversions between ligand conformations) cannot be resolved.

The experimental CCSs determined for different charge states for Au_10_(TGA)_10_ nanoclusters are given in Table 1. The collision cross-section for the three-charge state was only slightly higher by ~4% than that for the two-charge state. This finding is in contrast with Au_10_(SG)_10_, where a charge-induced unfolding due to Coulomb repulsion between charged moieties was observed, producing more dramatic effects on the CCS [18]. Indeed, for Au_10_(SG)_10_, the increase in the collision cross-section as a function of charge was more important (by ~6.5%). Furthermore, the size of the glutathione ligand was in the same order as the size of the metallic core. This indicates that the charging of the TGA ligand molecule played a minor role in the total collision cross-section of Au_10_(TGA)_10_. This means that the overall structure of the NCs was not significantly modified by the charge, as confirmed by DFT structures obtained for neutral Au_10_(TGA)_10_ and [Au_10_(TGA)_10_−2H]^2−^ (see Figure S5 in the Supplementary Materials). For the two charge state, the CCS value calculated from the [5,5] and [6,4] catenane structures matched the experimental CCS value, confirming that core geometry was consistent with a catenane-like form for Au_10_(TGA)_10_ nanoclusters.

## 4. Conclusions

The chemistry of the sulfur–gold bond is extremely rich and leads to hybrid materials. Such materials encompass gold thiolate coordination oligomers, for instance Au*_n_*(SR)*_n_* and atomically well-defined clusters Au*_n_*(SR)*_m_*, or supramolecular assemblies like –(AuSR)_∞_–. The catenane-like structure is a unique feature of Au*_n_*(SR)*_n_* complexes, but certainly also in thiolate-protected metal nanoclusters at a low Au/SR ratio limit (i.e., approaching 1:1). Unraveling the total structure of gold nanoclusters is of paramount importance for their characterization. Unfortunately, the use of X-ray crystallography is problematic for homoleptic thiolate-protected metal nanoclusters, because sample crystallization requires extremely high purity and stability. Additional characterization tools able to distinguish structural isomers are thus highly desirable. The DFT approach provides information about catenane-like structures for the two lowest energy isomers. The TDDFT absorption features allows for the structural assignment to experimental data, as well. Ion mobility-mass spectrometry (IM-MS) has proven to be a useful complement to MS due to its ability to separate ions based on their “shape”. In this work, we used this coupling and additionally reported collision cross-sections (CCS) for selected gas phase charge states of Au_10_(TGA)_10_ cluster ions. Charge effects on the CCS were found negligible for a simple and small thiolated ligand (thioglycolic acid (TGA)). Furthermore, the comparison of CCS values from different structural isomers of Au_10_(TGA)_10_ obtained at the DFT level of theory has permitted confirming the catenane structure for such nanoclusters.

## Figures and Tables

**Table 1:** Experimental and calculated collision cross-section (CCS) values for three isomers of Au_10_(TGA)_10_ NCs are given. The influence of charge has been experimentally determined (error bars are in brackets). For this purpose, the trajectory method has been used [27]. The DFT structures obtained for [Au_10_(TGA)_10_−2H]^2−^ are given in Figure S5 in the Supplementary Materials.

CCS of Au_10_(TGA)_10_ (Å^2^)	Au_10_(TGA)_10_ neutral	[Au_10_(TGA)_10_−2H]^2−^	[Au_10_(TGA)_10_−3H]^3−^
Exp.		225 (5)	235 (5)
[5,5] catenane	212	220	
[6,4] catenane	228	230	
Crown-like	196	196	

## Supplementary Materials

The following are available online at https://www.mdpi.com/2079-4991/9/3/457/s1: Figure S1: Chemical structure of thioglycolic acid (TGA). Figure S2: Collision-induced dissociation spectra of (Au_10_(TGA)_10_)^3−^. Figure S3: ATDs recorded for two charge states of Au_10_(TGA)_10_ in negative mode. Figure S4: ATDs recorded for two charge states of Au_10_(TGA)_10_ compared to the fick law. Figure S5: DFT structures obtained for [Au_10_(TGA)_10_−2H]^2−^.
