# Supplementary material for: Catenane Structures of Homoleptic Thioglycolic Acid-Protected Gold Nanoclusters Evidenced by Ion Mobility-Mass Spectrometry and DFT Calculations
Source: Nanomaterials (Basel). 2019 Mar 19;9(3):457. doi: 10.3390/nano9030457 (PMC6474107; doi:10.3390/nano9030457)
Supplement: Supplementary file 1 [file nanomaterials-09-00457-s001.pdf]

# Supplementary Materials

# Catenane Structures of Homoleptic Thioglycolic Acid-Protected Gold Nanoclusters Evidenced by Ion Mobility-Mass Spectrometry and DFT Calculations

**Clothilde Comby-Zerbino [1], Martina Perić [2], Franck Bertorelle [1], Fabien Chirot [3], Philippe Dugourd [1], Vlasta Bonačić-Koutecký [2,4] and Rodolphe Antoine [1,*]**

[1] Institut Lumière Matière UMR 5306, Université Claude Bernard Lyon 1, CNRS, Univ Lyon, F-69100, Villeurbanne, France; clothilde.zerbino@univ-lyon1.fr (C.C.-Z.); franck.bertorelle@univ-lyon1.fr (F.B.); philippe.dugourd@univ-lyon1.fr (P.D.)

[2] Center of Excellence for Science and Technology-Integration of Mediterranean region (STIM) at Interdisciplinary Center for Advanced Sciences and Technology (ICAST), University of Split, Poljička cesta 35, HR-21000 Split, Croatia; martina@stim.unist.hr (M.P.); vbk@stim.unist.hr (V.B.-K.)

[3] Institut des Sciences Analytiques UMR 5280, Université Claude Bernard Lyon 1, ENS de Lyon, CNRS, Univ Lyon, 5 rue de la Doua, F-69100, Villeurbanne, France; fabien.chirot@univ-lyon1.fr

[4] Department of Chemistry, Humboldt Universitat zu Berlin, Brook-Taylor-Strasse 2, 12489 Berlin, Germany

[*] Correspondence: rodolphe.antoine@univ-lyon1.fr; Tel.: +33-472-43-1085

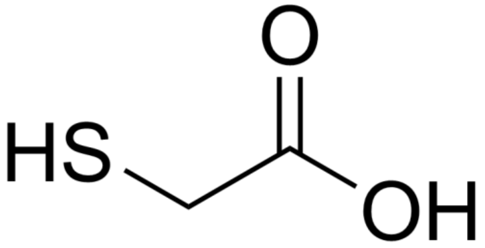


**Figure S1.** Chemical structure of thioglycolic acid (TGA).

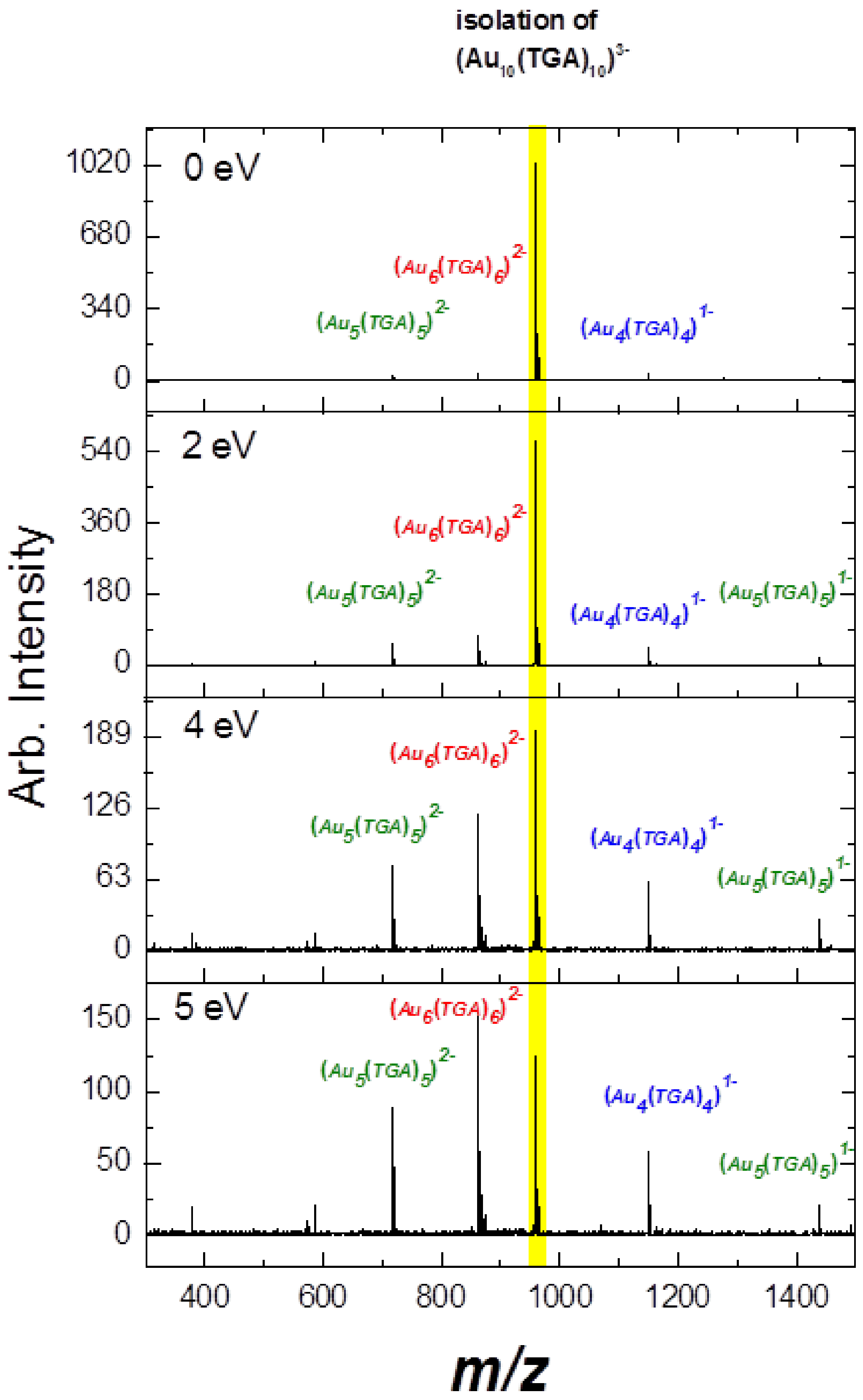


**Figure S2.** Collision-induced dissociation spectra of $(Au_{10}(TGA)_{10})^{3-}$. $(Au_{10}(TGA)_{10})^{3-}$ is selected with quadrupole (m/z: 959 ± 10) and fragmented with different collision energy in the collision cell.

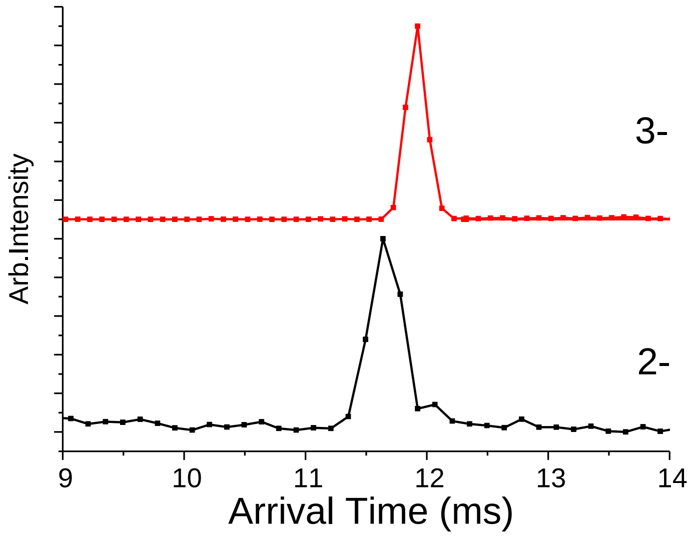


**Figure S3.** Arrival time distributions recorded for two charge states of $Au_{10}(TGA)_{10}$ in negative mode: 3- (red line) and 2- (black line). Drift conditions for the drift tube: 4.0 Torr helium at 296 K, drift voltage 450 V. The width of the peaks observed is resolution-limited (0.3 ms).

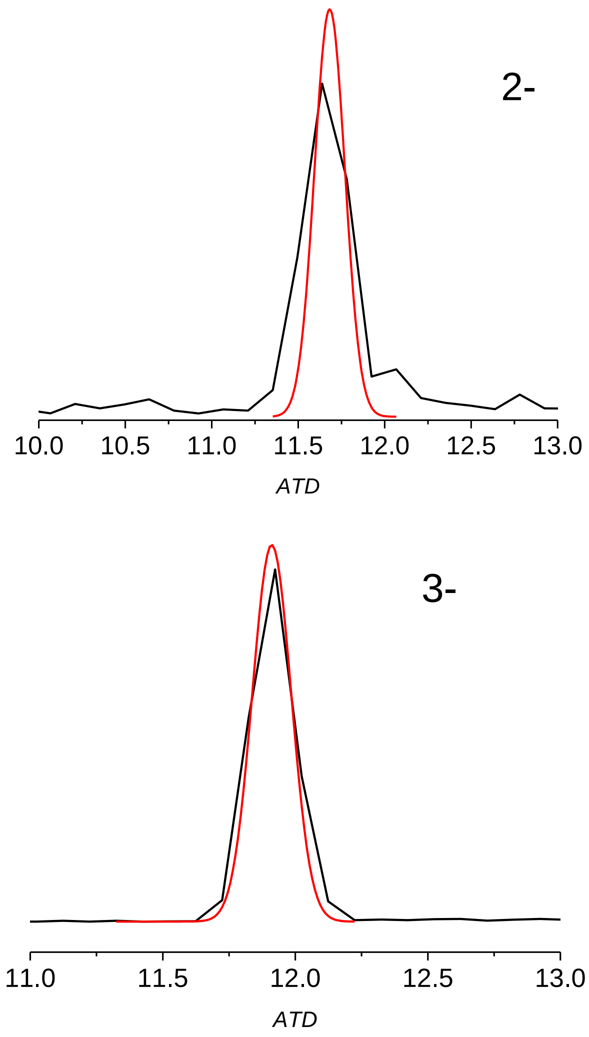


**Figure S4.** Arrival time distributions (ATDs) recorded for the charge states of $Au_{10}(TGA)_{10}$ in negative mode: 3- and 2- with the corresponding ATDs expected by the Fick law.

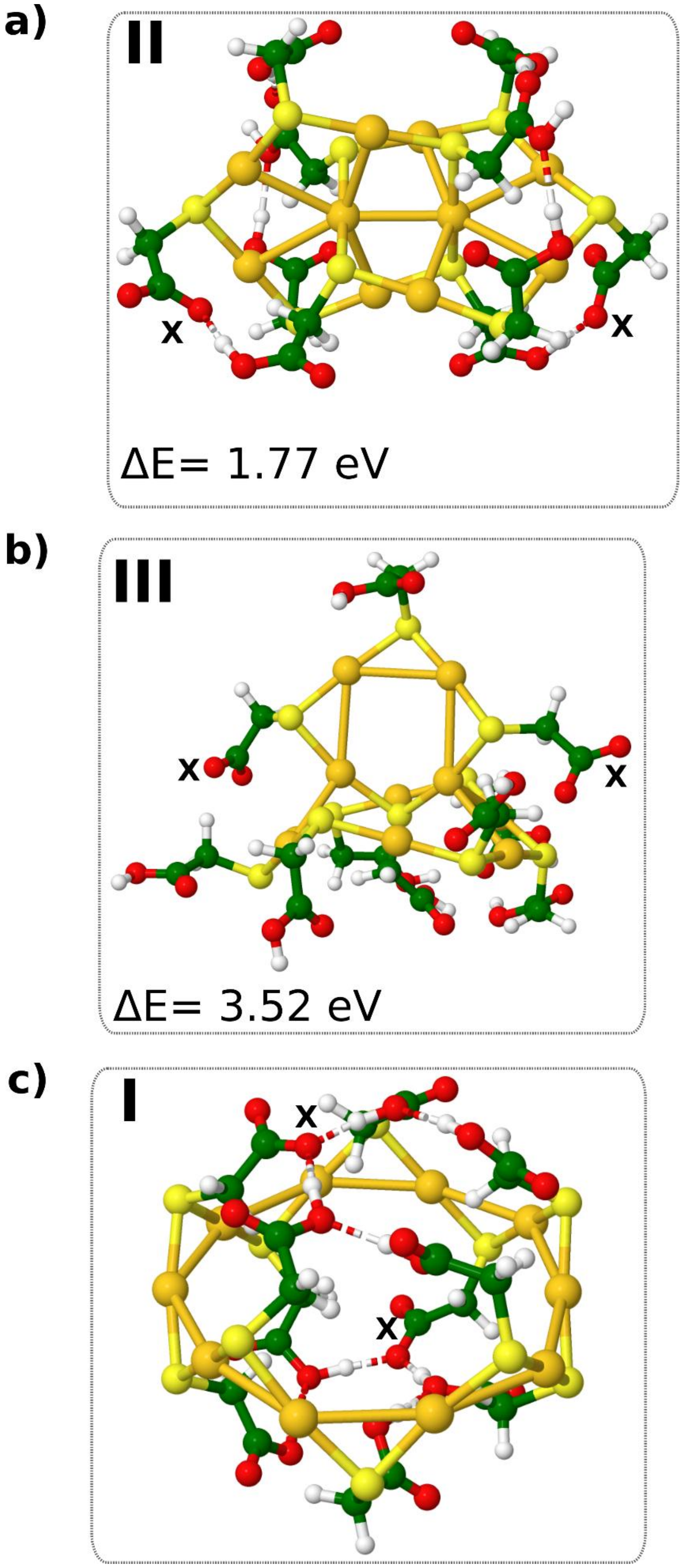


**Figure S5.** Calculated structures obtained for $[Au_{10}(TGA)_{10}{-}2H]^{2-}$. X corresponds to the deprotonation of the carboxylic group. (**a**) Isomer II, (**b**) isomer III and (**c**) isomer I.
